# Effects of hydrogel stiffness and viscoelasticity on organoid culture: a comprehensive review

**DOI:** 10.1186/s10020-025-01131-7

**Published:** 2025-03-03

**Authors:** Wei Lai, Hu Geliang, Xu Bin, Wei Wang

**Affiliations:** 1 Department of Thoracic Surgery, Renmin Hospital of Wuhan University, Wuhan, 430060 China; 2 Department of Orthopedics, Renmin Hospital of Wuhan University, Wuhan, 430060 China; 3 Cancer Center, Renmin Hospital of Wuhan University, Wuhan, 430060 China

**Keywords:** Organoids, Stiffness, Viscoelasticity, 3D model, Hydrogel

## Abstract

As an emerging technology, organoids are promising new tools for basic and translational research in disease. Currently, the culture of organoids relies mainly on a type of unknown composition scaffold, namely Matrigel, which may pose problems in studying the effect of mechanical properties on organoids. Hydrogels, a new material with adjustable mechanical properties, can adapt to current studies. In this review, we summarized the synthesis of recent advance in developing definite hydrogel scaffolds for organoid culture and identified the critical parameters for regulating mechanical properties. In addition, classified by different mechanical properties like stiffness and viscoelasticity, we concluded the effect of mechanical properties on the development of organoids and tumor organoids. We hope this review enhances the understanding of the development of organoids by hydrogels and provides more practical approaches to investigating them.

## Introduction

Extracellular matrix (ECM), studied on three-dimensional (3D) microenvironments for many years, are now recognized as not only serving as structural support for cells to reside but also providing diverse biochemical and biophysical cues (Geiger et al. 2009). For example, the ECM provides diverse cell adhesion ligands to specifically bind cell surface receptors (typically integrins), forming focal adhesions or hemidesmosome responses to different mechanical cues (Revach et al. 2020). Such cell-ECM play essential roles in cell survival, spreading, proliferation, migration, and differentiation (Kanchanawong and Calderwood 2023). Similar to cell surface receptors, mechanical features of the extracellular matrix (ECM), such as the organization of type I collagen fibers, play a crucial role in directing the orientation and migration of various cell types (Liu et al. 2019). According to remodeling ECM, mechanical properties, including increased/decreased stiffness or changing viscoelasticity, can also promote tumorigenesis (Mohan et al. 2020). Therefore, mimicking the mechanical property of ECM is of great significance for investigating the behaviors of cells.

Many studies have concentrated on determining how to change stiffness or viscoelasticity to regulate tissue response (Mohammadi and Sahai 2018). For example, the ECM can provide growth factors and modulate tissue stiffness, such as wounded tissue, by disrupting the basement membrane (Lou and Mooney 2022; Wilson et al. 2020). Controlling stiffness can affect stem cell differentiation, which was confirmed in 2017 (Vining and Mooney 2017). Moreover, increasing stiffness can also promote diseases, such as breast tumors, due to excessive deposition and crosslinking of collagen (Patwardhan et al. 2021). However, for the development and regeneration of certain healthy tissues, stiffer hydrogels are required as ECM scaffolds (Zanut et al. 2023). Therefore, studying the mechanical properties of the ECM is critical for understanding cell development and disease pathogenesis. Studying ECM requires 3D cell models that can enable the study of more complex 3D interactions, which 2D cell models cannot provide (Sailer et al. 2023). To reconstruct the effects of ECM on cells in vivo, researchers have developed 3D cell models derived from self-organizing stem cells called organoids (Zhu et al. 2023a). These organoids can recapitulate the original tissues’ in vivo architecture, functionality, and genetic signature (Dutta et al. 2017). However, creating an ECM-mimicking environment is challenging due to the complexity and dynamic characteristics of the ECM. Matrigel, derived from the Englbreth-Holm-Swarm mouse sarcoma, has been extensively used as a 3D cell culture material for many years (Kleinman and Martin 2005). Prior to Matrigel, hydrogels were recognized for their non-toxic biological properties and adjustable mechanical properties, including stiffness and viscoelasticity, and are thus considered ideal properties for investigating the role of ECM mechanical properties in organoid development (Cao et al. 2021; Chaudhuri et al. 2020).

Currently, research on tumor models primarily encompasses two-dimensional cell cultures, patient-derived xenograft models, and tumor organoid models. However, due to the complex composition of tumors, which cannot be determined by a single factor or cell type, two-dimensional cell culture models fail to reflect the true conditions within tumors fully. Patient-derived tumors transplanted into immunodeficient mice preserve the heterogeneity of tumor-stroma interactions. The tumor microenvironment gradually becomes replaced by murine-derived cells, leading to differential differentiation trajectories of tumor cells (Yang et al. 2023). Constructing organoid models provides a system independent of the local microenvironment for studying specific tissues. Additionally, we can modulate organoid development by altering molecular, cellular, tissue, and system parameters (stiffness and viscoelasticity), offering advantages that traditional models cannot match (Jin et al. 2024). Consequently, organoid technology has made significant advances in studying of typical physiological structures, biological behaviors, disease occurrence, and drug development in recent years, with the potential to replace animal models in medical research (Han et al. 2024; Zhang et al. 2024).

This review summarizes current methods for synthesizing hydrogels with tunable stiffness and viscoelasticity, focusing on techniques to control these mechanical properties. Recent studies have shown that manipulating the mechanical properties of hydrogels can significantly influence organoid morphology under identical culture conditions. Therefore, we elucidate the differentiation trajectories of organoids in response to varying mechanical environments. Additionally, we emphasize the impact of material biology factors on cell culture and organoid formation, offering insights for the development of novel hydrogels. Finally, we explore the potential applications of various hydrogels in organoid culture.

## Application of hydrogels with adjustable stiffness in organoids

**Fig. 1:**
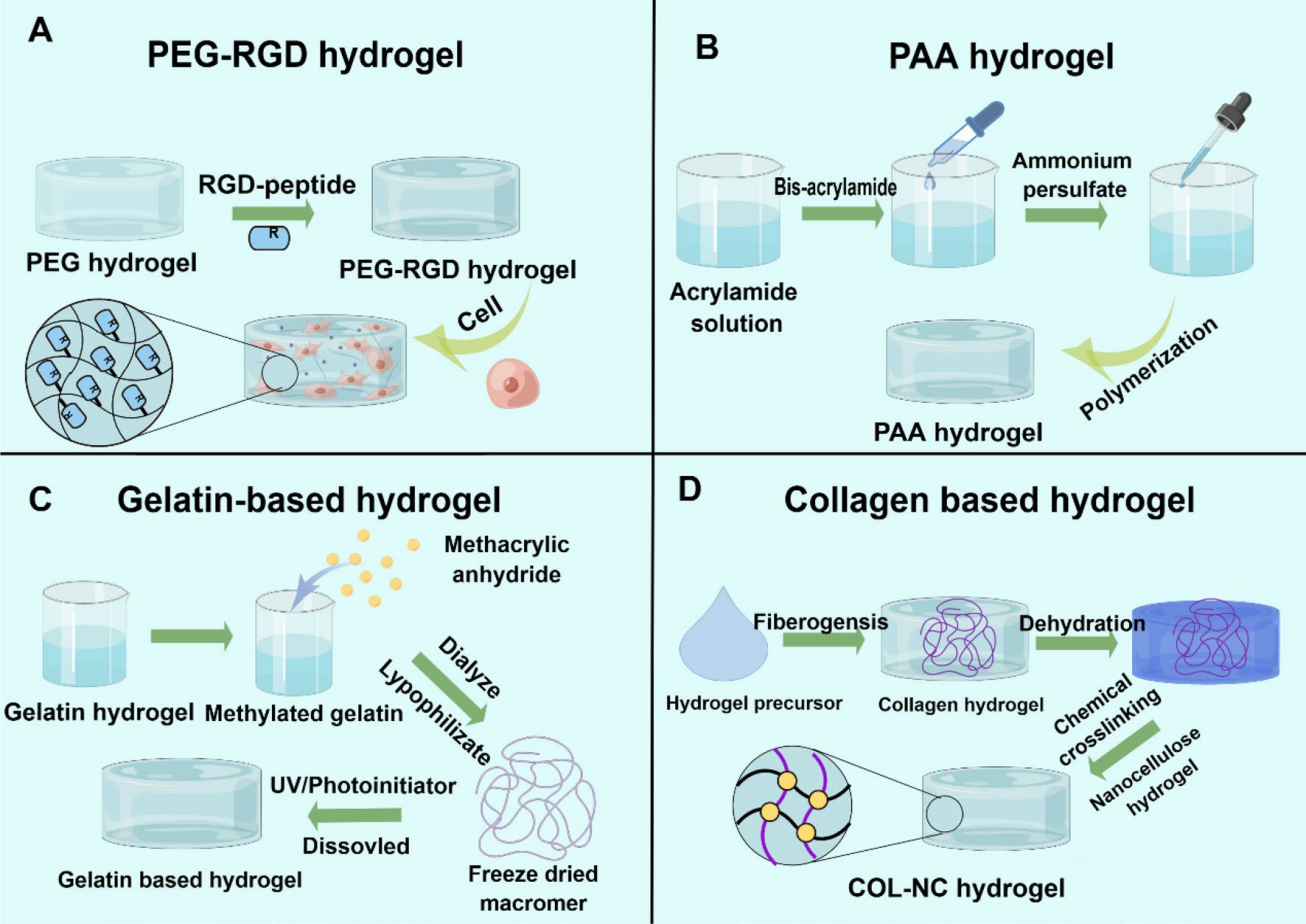
The synthesis of hydrogels with adjustable stiffness. **A** Synthesis of PEG-RGD hydrogel. PEG hydrogels, widely used for their biological stability in cell culture, require the addition of RGD sequences to facilitate cell adhesion. The stiffness of PEG-RGD hydrogels can be modified by adjusting the PEG content. **B** Synthesis of PAA hydrogel. PAA, as a synthetic hydrogel, exhibits a wide range of stiffness (2 Pa to 55 kPa). Specifically, acrylamide and bis-acrylamide can be polymerized into polyacrylamide (PAA) hydrogel within minutes using TEMED and ammonium persulfate. **C** Synthesis of gelatin-based hydrogel. Methacrylic anhydride is reacted with gelatin in phosphate-buffered saline (PBS) under vigorous stirring. The mixture is then dialyzed against PBS and lyophilized to obtain the macromer. The hydrogel is formed by incorporating Irgacure as a photoinitiator. **D** Synthesis of collagen-based hydrogel. After synthesizing the collagen hydrogel precursor, a chemical crosslinking method is used to combine it with NC hydrogel to form the COL-NC hydrogel. The hydrogel incorporates NC fibers, which enhance its stiffness. By adjusting the NC hydrogel content, hydrogels with varying stiffness can be synthesized. Created by figdraw

Previous research has shown that the stiffness and degradation of ECM can promote cancer cell proliferation by transforming the growth factor-β (TGF-β) related pathway, while ECM degradation through the matrix metalloproteinase (MMP) pathway can facilitate tumor microenvironment invasion (Najafi et al. 2019). Hydrogels are soft, water-swollen materials with a three-dimensional porous network structure, composed of polymeric molecules, fibers, or particles, in which water or aqueous phases serve as the dispersing medium (Mamidi et al. 2024). Due to their high-water content, they exhibit biocompatibility and structural similarities to the native ECM, making them highly suitable for engineering cell microenvironments (Bertsch et al. 2023; Chooi et al. 2023). Prior to the work of Matrigel, the role of hydrogels was primarily known by their biological non-toxic, and adjustable mechanical properties (Zhang et al. 2023a). In this part, we summarize the synthesis of controlled hydrogels and discuss their application in organoids with adjustable stiffness (Fig. 1).

### Classification of hydrogels with adjustable stiffness

Hydrogels can be classified in various ways; however, the classifications discussed in this paper are among the most commonly recognized, based on source, crosslinking method, and controllable mechanical properties.

#### Source

Natural hydrogels composed of proteins and polysaccharides are highly compatible with tissue formation, cell growth, and phenotype maintenance, but they suffer from poor mechanical properties and uncontrolled degradability (Jiang et al. 2020; Lin et al. 2022; Xia and Chen 2022). Recent studies have developed hydrogels that combine two different natural hydrogels. For instance, Curvello and colleagues developed a new hydrogel crosslinking approach by combining two natural hydrogels in 2021 (Curvello et al. 2021). As previously reported, they synthesized NC hydrogels using TEMPO-periodate oxidation and then mixed them with COL hydrogels to create the COL-NC hydrogel (Mendoza et al. 2020). This hydrogel includes NC fibers that help increase the material’s stiffness and pressure resistance. In addition, numerous natural hydrogels have been outlined in detail in previous reviews (Ho et al. 2022; Hu et al. 2023; Zhu et al. 2023b).

Compared to natural hydrogels, synthetic hydrogels can be prepared in large quantities and manipulated at the molecular level through polymerization, crosslinking, and functionalization (Cao et al. 2021; Pahapale et al. 2022; Hushka et al. 2020). For instance, acrylamide and bis-acrylamide can be polymerized with TEMED and ammonium persulfate to create polyacrylamide (PAA) hydrogels in just a few minutes (Yeung et al. 2005). In order to enhance cell adhesion, RGD peptide (Ac-GCGYG**RGD**SPG-NH2) can be added to the hydrogel (Ogle et al. 2020; Martello et al. 2014). Synthetic hydrogels exhibit a wide range of stiffness, with PAA hydrogels ranging from 2 Pa to 55 kPa, which is suitable for most organoids (Yeung et al. 2005; Yavitt et al. 2022; Leonard-Duke et al. 2023). Recently, composite hydrogels that combine the characteristics of both natural and synthetic hydrogels have been developed (Sun et al. 2021; Hu et al. 2021; Greco et al. 2024a). These composite hydrogels have been employed in the development of intestinal organoids. For instance, methacrylic anhydride is reacted with gelatin in phosphate-buffered saline (PBS) under vigorous stirring, followed by dialysis against PBS and subsequent lyophilization to obtain the macromer. The gel is then formed by incorporating irgacure as a photoinitiator (Nichol et al. 2010).

#### Crosslinking method

Hydrogels have recently emerged as promising materials in organoids due to their unique characteristics of spatial dynamics and mechanical, resulting from reversible linkages (Curvello et al. 2021; Hushka et al. 2020; Yeung et al. 2005; Nichol et al. 2010). These gels can be classified as physical or chemical gels based on crosslinking methods, which provide dynamic mechanical support to the structure (Table 1). Physical hydrogels exhibit dynamic properties as the interactions between polymer chains are not permanent but strong enough to maintain the integrity of the gel in water. Chemical hydrogels are created by two different gel-forming techniques: free radicals to initiate crosslinking and a reaction between active functional groups on the polymer chain to form covalent bonds, resulting in a stable gel structure in water. For example, Gan et al. (2021) employed the cholesterol-modified non-coding microRNA (Chol-miR-26a) to enhance the osteogenic differentiation of human mesenchymal stem cells. This PEG-based hydrogel can be modified by poly (lactide-co-glycolide) (Yang et al. 2020a; Ge et al. 2018), polyvinyl alcohol (Wan et al. 2015) and glycol diacrylate (Liu et al. 2021) to form injectable temperature-controlled hydrogels. Additionally, Stengelin (Stengelin et al. 2020) et al. introduced a cross-linked transglutaminase system in the compound hydrogels of PEG and HA to form bone marrow organoids. The new strategy optimized the physical and biological properties of this hydrogel system via combining PEG and HA to form the bone marrow organoids. This work demonstrated that the TG-PEG/HA hybrid hydrogels were beneficial in maintaining the morphology, proliferation, or differentiation of human bone marrow-derived stromal cells and human hematopoietic and progenitor stem cells in vitro*.* Hydrogels possess exceptional organoid research properties, highlighting their versatility and potential (Chen et al. 2023).

**Table 1:** The application of hydrogels with adjustable stiffness in the development organoid

Hydrogel	Crosslinking methods	Mechanical control	Organoid	Function	Stiffness	Reference
PEG hydrogel	Chemical crosslinked	Concentration	Neural tube	Polarization	2 kPa	Abdel Fattah et al. (2021)
PEG-based hydrogel	Physically crosslinked	Photodegradation	Intestinal	Crypt formation	1.3 kPa	Hushka et al. (2020)
PEG-based hydrogel	Physically crosslinked	Concentration	Liver	Differentiation	1.3 kPa	Sorrentino et al. (2020)
PEG-based hydrogel	Physically crosslinked	Concentration	Cerebellar	Development	61.2 kPa	Balion et al. (2020)
PAA hydrogel	Chemical crosslinked	Concentration	Intestinal	Colony survival	0.7 kPa	Pérez-González et al. 2021)
PAA hydrogel	Chemical crosslinked	Concentration	Kidney	Differentiation	1 kPa	Garreta et al. (2019)
Collagen-based hydrogel	Physically crosslinked	Concentration	Intestinal	Crypt formation	90 Pa	Curvello et al. (2021)
PEG-based hydrogel	Chemical crosslinked	Concentration	Bone	Osteogenic differentiation	5663 Pa	Bi et al. (2019)
PEG-based hydrogel	Chemical crosslinked	Concentration	Bone	Adipogenic differentiation	0.6 kPa	Frith et al. (2018)
DNA-SF hydrogel	Chemical crosslinked	DNA concentration	Cartilage	Cartilage differentiation	30.2 kDa	Zhou et al. 2024)

#### Controllable mechanical properties

Hydrogels can be classified into two types: conventional hydrogels with no or limited control over their mechanical properties and controllable hydrogels that allow for adjustable mechanical properties. Controllable hydrogels can alter their mechanical properties through various methods. For example, PEG-based hydrogels can change their stiffness by adjusting the concentration of PEG. By varying the PEG concentration to 2.5, 4.5, and 6.5% w/v, the hydrogel stiffness can be soft (0.5 kPa), intermediate (3 kPa), or stiff (5.5 kPa), respectively (Funfak et al. 2019). Similarly, hydrogels with different stiffness can be obtained by reacting PEG with chloroacetyl chloride (PEGdA). To achieve different stiffness, the PEGdA concentration can be adjusted. For instance, PEGdA concentrations of 4%, 6%, and 8% can produce hydrogel stiffness of 2.27, 9.99, and 25.23 kPa, respectively (Cho et al. 2009). Furthermore, by varying the type of photoinitiator and combining it with different hydrogels, the stiffness of PEGdA hydrogels can also be modulated (Hakim Khalili et al. 2023). This range of stiffness corresponds to the stiffness of the liver and has been used in developing liver organoids (Lee et al. 2020, 2017). In recent years, a new hydrogel crosslinked with tetrafunctionalized PEG dibenzocyclooctyne (PEG-DBCO) and allyl sulfide bis-azide can manipulate the hydrogel’s modulus in a precise and predictable manner. Under 365 nm light degradation, this hydrogel can generate a range of soft conditions ranging from 500 Pa to 1.3 kPa, making it an ideal material for studying crypt formation in intestinal organoids (Hushka et al. 2020).

There are alternative hydrogels with adjustable mechanical properties beyond the widely used PEG hydrogels. The stiffness of PAA hydrogels can be adjusted by varying the concentrations of acrylamide and bis-acrylamide. Soft hydrogels (1 kPa) contain 5% acrylamide and 0.04% bis-acrylamide, while stiffer hydrogels (60 kPa) contain 12% acrylamide and 0.25% bis-acrylamide (Garreta et al. 2019). PAA gels have a broad range of stiffness from 2 Pa (3% acrylamide and 0.05% bis-acrylamide) to 55 kPa (12% acrylamide and 0.60% bis-acrylamide), which is suitable for most organoids (Lee et al. 2017). Similarly, the stiffness of gelatin-based hydrogels can be altered by adjusting the methacrylate degree. By changing the methacrylate amount used for the reaction, hydrogels with different stiffness can be obtained. For instance, a softer hydrogel (2% v/v) has a stiffness of 1.5 kPa, while a stiffer hydrogel (8% v/v) has a stiffness of 1.5 kPa (Yue et al. 2018). This degree of methylation has been used to study breast tumors (Prince et al. 2022). Besides, collagen-based hydrogels can be made with different c nanocrystalline (NC) fiber concentrations to achieve different stiffness. The stiffness of a collagen-based hydrogel with 0.3 wt% NC fibers is higher than that of a hydrogel with 0.2 wt% NC fibers. Alternatively, different gelatin methylation degrees can be crosslinked with 4-arm PEG acrylate to achieve different stiffness (Casey et al. 2017).

Developing functional and biomimetic materials to engineer hydrogels with adjustable stiffness has provided a strong foundation for various technological advancements. In this part, we summarize the synthesis of hydrogels that have been utilized for developing organoids with adjustable stiffness. However, challenges such as the potential for contamination with organoid materials during hydrogel synthesis remain to be addressed. Furthermore, we discuss the various methods used to modify stiffness, including adjustments to concentrations and exposure to UV light. While numerous hydrogels have been developed, new hydrogels with more consistent and reliable stiffness remain a crucial challenge for further progress in this field.

### Utilizing hydrogels with adjustable stiffness for influencing organoid development

**Fig. 2:**
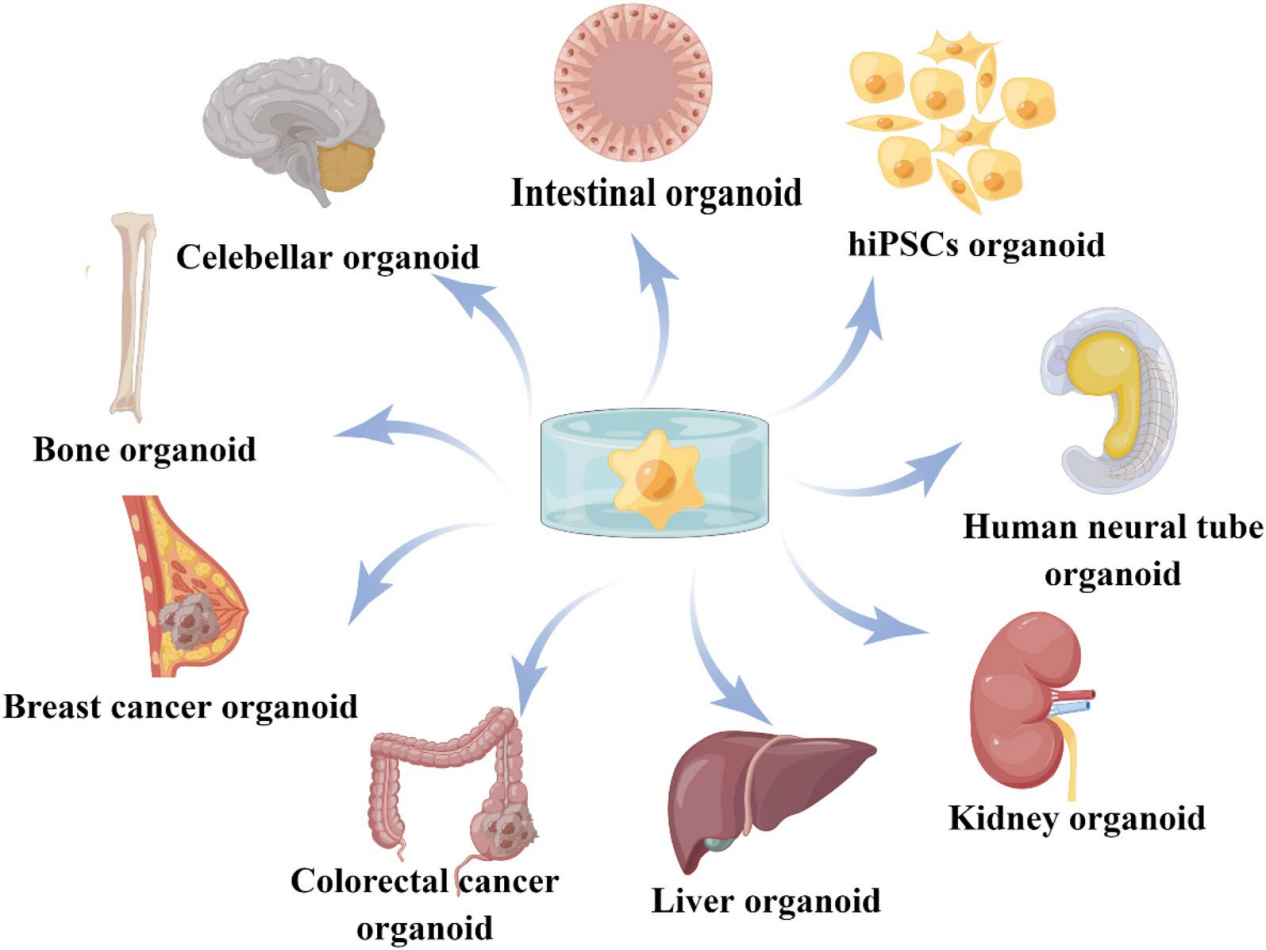
Utilization of hydrogel with adjustable mechanical properties in organoids. Hydrogels with adjustable stiffness have already been used to generate intestinal organoids that can be studied for crypt formation and development. Hydrogels with adjustable stiffness have further been used to study related behaviors of kidney organoids, human neural tube organoids, and liver organoids. Created by figdraw

Over the past decades, most research on organoids has emphasized the use of Matrigel, which has been widely regarded as the “gold standard” scaffold for cell growth in vitro (Li and Kumacheva 2018; Hill et al. 2021). However, due to various undefined components of Matrigel, it is hard to study how ECM stiffness affects organoid behavior by controlling its mechanical properties (Habanjar et al. 2021; Hong et al. 2023). Recently, more and more attention has been focused on providing hydrogel, a new material with adjustable stiffness (Ting et al. 2021; Zhang et al. 2022). Much literature has been published on applying hydrogels with adjustable stiffness in organoid development (Meran et al. 2023). In this section, we summarize current research on organoid development in adjustable hydrogels with the hope of inspiring new ideas for future studies. (Fig. 2).

#### Intestinal organoid

To date, numerous studies have investigated how altering the microenvironmental stiffness of intestinal organoids can influence their behaviors, including crypt size (Pérez-González et al. 2021), colony formation (Gjorevski et al. 2016), and viability (Curvello et al. 2021). For example, studies have developed intestinal organoids on different stiffness substrates (ranging from 0.2 to 15 kPa) to investigate the effects of stiffness on crypt size, revealing that an increase in substrate stiffness leads to a decrease in the size of the stem cell compartment and the number of stem cells therein due to the inability of the substrate to facilitate proper crypt folding (Pérez-González et al. 2021). On the other hand, intestinal stem cells grown in a stiffness of 1.3 kPa and soft matrices (300 Pa) exhibit poor expansion and proliferation compared to those living in 1.3 kPa stiffness (Gjorevski et al. 2016). Interestingly, stiffness can elicit different effects on intestinal organoids at different stages of development. For example, a stiffness of 1.3 kPa is optimal for colony survival, whereas a higher or lower modulus results in a decreased number of living colonies (Hushka et al. 2020). In contrast, when forming crypts, a softer substrate (0.2 kPa) is preferable for crypt formation compared to the most suitable stiffness of 1.3 kPa for colony survival (Pérez-González et al. 2021). YAP/Notch signaling has emerged as a novel mechanosensitive pathway, identified as a critical player in both epithelial organization and crypt maintenance. He et al. demonstrated that substrate stiffness modulates the differentiation properties of intestinal stem cells (He et al. 2023). Increased stiffness enhances cytoplasmic YAP expression, promoting the extension of olfactomedin-4+ cells into the villus region, while concurrently inducing nuclear translocation of YAP. This shift results in preferential differentiation of intestinal stem cells into goblet cells, ultimately contributing to disease development. Therefore, when studying the behaviors of intestinal organoids, it is essential to consider different stiffness substrates. Future studies could explore and compare YAP/Notch signaling in different mechanical environments to better understand organoid dependence on local matrix mechanics.

#### Liver organoid

**Fig. 3:**
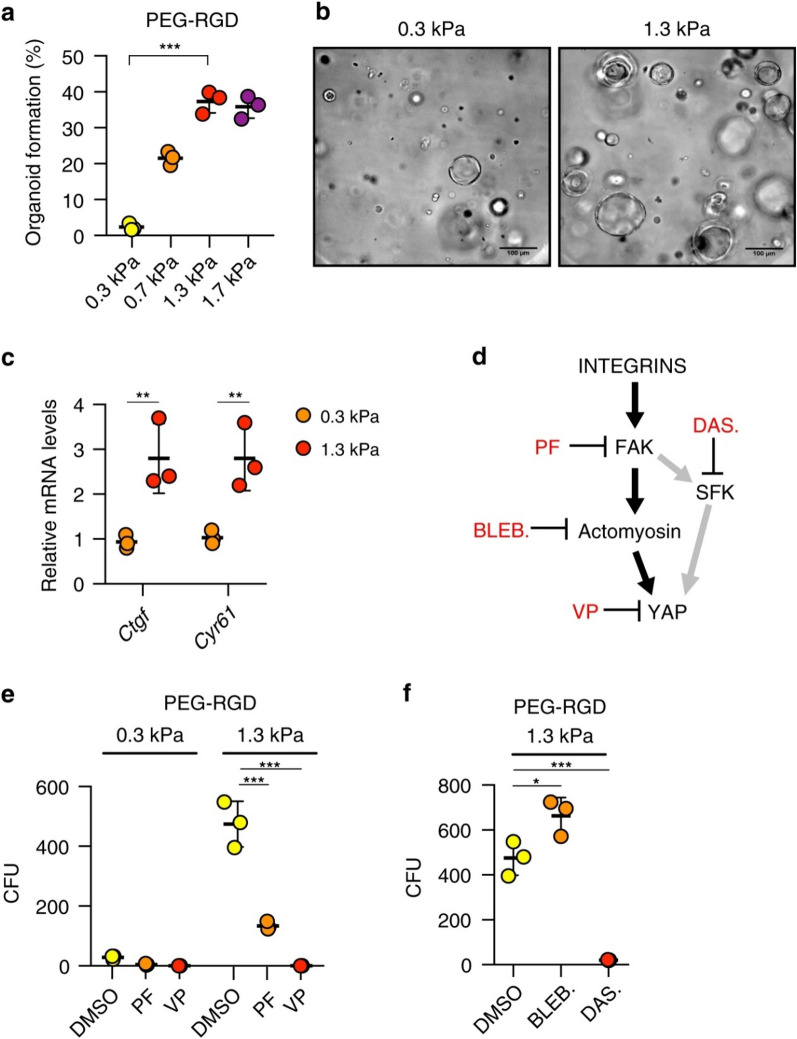
**a** Effect of matrix stiffness on organoid formation efficiency. Graphs show individual data points derived from *N* = 3 independent experiments and means ± s.d. (*P* < 0.0001). **b** Representative image of organoids 3 days after embedding in PEG-RGD hydrogels of indicated stiffness. **c** Gene expression was analyzed by qRT-PCR in liver organoids 6 days after embedding in PEG-RGD hydrogels with indicated stiffness. Graphs show individual data points derived from *N* = 3 independent experiments and means ± s.d. (*P* = 0.0088; 0.0077). **d** Schematic representation of cellular mechano-signaling pathways. Inhibitors of key elements are depicted in *red*. **e** Effect of indicated inhibitors on organoid formation efficiency in soft (300 Pa) and physiologically stiff (1.3 kPa) PEG-RGD hydrogels. CFU (colony forming unit). Graphs show individual data points derived from *N* = 3 independent experiments and means ± s.d. (*P* < 0.0001). **f** Effect of indicated inhibitors on organoid formation efficiency in physiologically stiff (1.3 kPa) PEG-RGD hydrogels. CFU (colony forming unit). Graphs show individual data points derived from *N* = 3 independent experiments and means ± s.d. (*P* = 0.0198; 0.0003). *P < 0.05, **P < 0.01, ***P < 0.001 one-way Anova (**a**, **f**) or two-way Anova (**c**, **e**). The image is from Sorrentino et al. (2020) and is reproduced with permission

Liver-specific ECM can be derived from surgically resected portion of damaged human livers or from non-transplantable livers. Lin and colleagues reported that their approach to liver tissue decellularization supported the growth and maintenance of rat hepatocytes; however, this method relied on mechanical disruption of the resected tissue, leading to the loss of organ architecture and vascular networks (Lin et al. 2004). In contrast, Jorke et al. developed an innovative ECM-derived hydrogel that exhibited minimal impact on gene or protein expression and demonstrated no species-specific effects between humans and pigs (Willemse et al. 2022). In addition, the mechanical properties of hydrogels can be adjusted to match the physiological stiffness of the liver. Giovanni Sorrentino et al. systematically described the role of hydrogel in liver organoids (Sorrentino et al. 2020). They compared PEG-based hydrogels (PEG, PEG-COL, PEG-FN, PEG-LAM, PEG-RGD) with Matrigel and identified that the PEG-based hydrogel could provide long-term organoid culture. Then, they identified liver organoids cultured in PEG hydrogel efficiently differentiate into hepatocyte-like cells, which shows this can mimic many of the established hepatic functions. To test whether matrix mechanics affects liver organoid growth, researchers grew liver organoids in hydrogels of variable stiffness, ranging from values below the standard liver stiffness (0.3 kPa) to those reaching physiological stiffness (1.3 kPa). This phenomenon is because stiffness has a mechano-sensitive (integrin-SFK-YAP) effect on liver organoid growth (Sorrentino et al. 2020). Clinical trials and laboratory tests have shown that normal liver stiffness plays a critical role in maintaining liver function. Monitoring changes in liver stiffness can serve as a predictive marker for mortality risk in patients with alcohol-related liver disease (Thorhauge et al. 2024). You et al. demonstrated that the activation of the TGF-β/Smad signaling pathway induces excessive synthesis of ECM, resulting in increased liver stiffness (You et al. 2020). Therefore, only the stiffness up to the physiological stiffness is optimal for formation. Higher or lower values can impair organoid formation (Fig. 3).

#### Kidney organoid

The normal human kidney comprises millions of filtering units called nephrons and is responsible for urine formation and blood filtration. Each nephron has three distinct compartments including glomerulus, a tubule and vasculature (Bülow and Boor 2019). Therefore, finding a material that can influence stem cell differentiation is important for culturing kidney organoid. Hydrogels with adjustable mechanical characteristics can mimic the ECM, which contains biological information that can be used to influence cellular responses and differentiation (Du et al. 2023). Employing hydrogels with different stiffness can induce pluripotent stem cell differentiation, which has been identified in kidney organoids (Garreta et al. 2019). In order to explore whether substrates mimicking a soft microenvironment may favor the generation of kidney organoids compared with stiffer substrates, Elena Garreta et al*.* developed pluripotent stem cells in soft hydrogel (1 kPa) and very rigid hydrogel (60 kPa). Compared to organoids grown on stiff matrices (60 kPa), pluripotent stem cells cultured on soft matrices (1 kPa) exhibit enhanced expression of kidney-related genes, potentially attributable to the reduced nuclear localization of YAP signaling. A soft milieu more accurately represents the early stages of embryonic development. During this time, counteraction gene regulatory networks control both pluripotency and differentiation ground states (Theunissen and Jaenisch 2017). Therefore, soft hydrogels are preferable to stiffer hydrogels as substrates for producing kidney organoids in a range.

#### Bone organoid and cartilage organoids

**Fig. 4:**
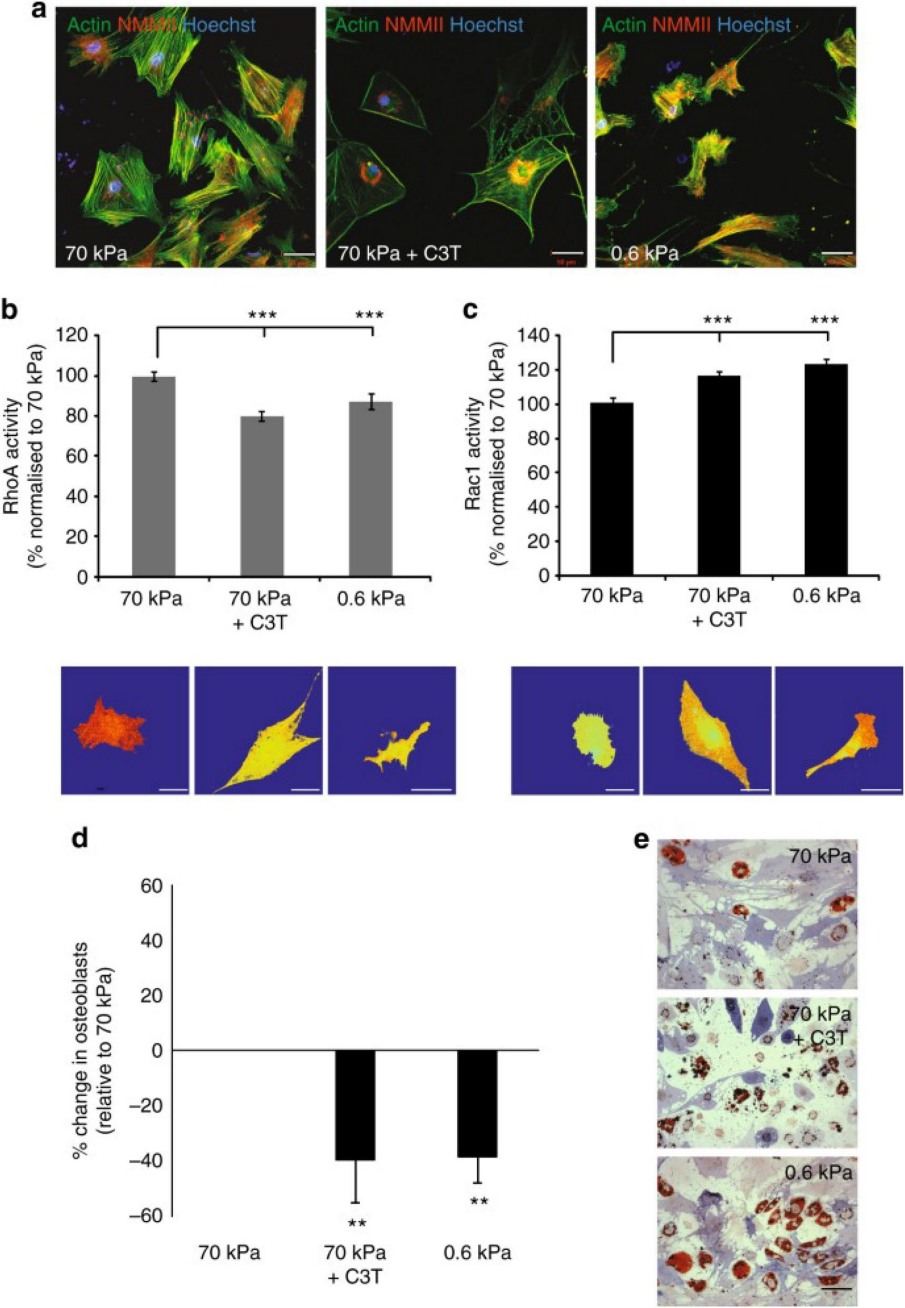
Characterization of hMSC properties in response to substrate stiffness and RhoA inhibition. **a** Morphology of hMSCs showing actin (*green*), nuclei (*blue*) and non-muscle myosin II (*red*). Scale bar, 50 μm. **B **RhoA and **c **Rac1 activity, as determined by FRET biosensor. Data is shown as mean ± SEM for hMSCs from three independent donors (*N* > 20/donor) with heatmaps of a representative cell. Scale bar, 50 μm. **D **Differentiation switch between osteogenesis and adipogenesis. Graph shows mean percentage change in osteoblasts ± SD for *N* = 4 MSC donors. **E **Representative images of alkaline phosphatase (*blue*) and Oil Red O (*red*) staining of hMSCs under the three different conditions. Scale bar, 100 μm. Samples were analyzed by one-way ANOVA with Tukey post hoc testing. Statistically different samples are denoted by *P < 0.05, **P < 0.01 and ***P < 0.001. The image is from Frith et al. (2018) and is reproduced with permission

In recent years, considerable research has been devoted to investigating the role of rigidity in regulating stem cell differentiation. Previous studies have indicated that mesenchymal stem cells (MSCs) can be induced to differentiate into adipocytes in a more flexible matrix, while they differentiate into osteoblasts in a rigid matrix. A notable contribution by Bi et al. involved the development of a functionalized hydrogel system using polyamidoamine (PAMAM) dendrimer and multi-armed thiolate PEG via thiol-ene Michael reaction, which allowed for precise control of stiffness (Bi et al. 2019). Their experiments found that the MSCs underwent osteogenic differentiation on the rigid hydrogel (5663 Pa) and adipogenic differentiation on the flexible hydrogel (77 Pa). These results were further supported by similar findings from other hydrogel systems (Frith et al. 2018; Pepelanova et al. 2018) (Fig. 3). Additionally, Darnell et al. conducted RNA studies on MSCs cultured in alginate hydrogels with varying moduli to gain insights into transcriptome-scale changes associated with rigidity detection (Darnell et al. 2018). Their work revealed a substantial number of genes that showed sensitivity to rigidity, indicating a non-linear relationship. Moreover, it has been reported that matrix rigidity can influence the osteogenic differentiation of cells through the activation of the mitogenic-activated protein kinase (MAPK) and signal-regulated extracellular kinases (ERK) pathway (Kyriakis and Avruch 2012). These findings suggest that MSCs cultured in soft hydrogels can maintain their stemness properties, while high-molecular-weight hydrogels with higher mechanical strength can induce chondrogenic differentiation of MSCs (Fig. 4). Furthermore, researchers have successfully designed and synthesized dual-network DNA-silk fibroin (DNA-SF) hydrogel that promotes cartilage repair in cartilage-like organs (Shen et al. 2024). Interestingly, unlike the previously reported 20 kPa (PEG) hydrogel, the silk-DNA hydrogel is most conducive to MSCs differentiation into chondrocytes at 30 kPa (Zhou et al. 2024). This phenomenon may be attributed to different signaling pathways activated by the hydrogel. DNA-SF hydrogels primarily upregulate the Wnt and TGF-β signaling pathways, thereby enhancing extracellular matrix (ECM) secretion and inducing chondrogenesis.

#### Human neural tube organoid

Previous studies have identified that mechanical forces play a particularly significant role in neurulation. However, it is still unclear how neural tissues integrate mechanical cues to coordinate fate specification with patterning, growth, and morphogenesis (Vijayraghavan and Davidson 2017). Recently, some studies focused on the effects of hydrogel stiffness on neural tube formation using 3D models (Abdel Fattah et al. 2021). By subjecting the human neural tube to mechanical stretch in hydrogels of varying stiffness, researchers revealed that the efficiency of floor plate patterning was only improved in intermediate stiffness (2 kPa) hydrogels. However, this phenomenon was not observed in soft (0.7 kPa) or stiff (8 kPa) hydrogels. Like floor plate patterning, a relative increase in size upon stretch is only evidenced at intermediate 2 kPa (Abdel Fattah et al. 2021). These results may be caused by the fact that organoid growth compresses the adjacent matrix, with the concomitant matrix resistance counteracting organoid growth. Therefore, neural tube organoids may use intermediate stiffness (2 kPa) hydrogel to study floor plate patterning and size factors.

#### Cerebellar organoid

Due to the recent development of cerebellar organoids in vivo, limited research has been conducted on this topic (Ballabio et al. 2020). However, the effect of stiffness on cerebellar organoid development has been studied (Balion et al. 2020). Balion et al. synthesized hydrogels with varying stiffness (80.1 and 61.2 kPa) and evaluated the expression of genes relevant to development. Their results showed that organoids grown on the 61.2 kPa hydrogel grew faster and more effectively than those on the 80.1 kPa hydrogel. Notably, the stiffness of these hydrogels falls within the range of stiffness measured for brain tissues and is at least four orders of magnitude lower than that of polystyrene (Cheng et al. 2008; Jiang and Xie 2019). This suggests that a lower level of stiffness may be necessary for optimal cerebellar growth, although further research is needed to determine the ideal conditions.

### The application of hydrogel with adjustable stiffness in tumor organoids

**Fig. 5:**
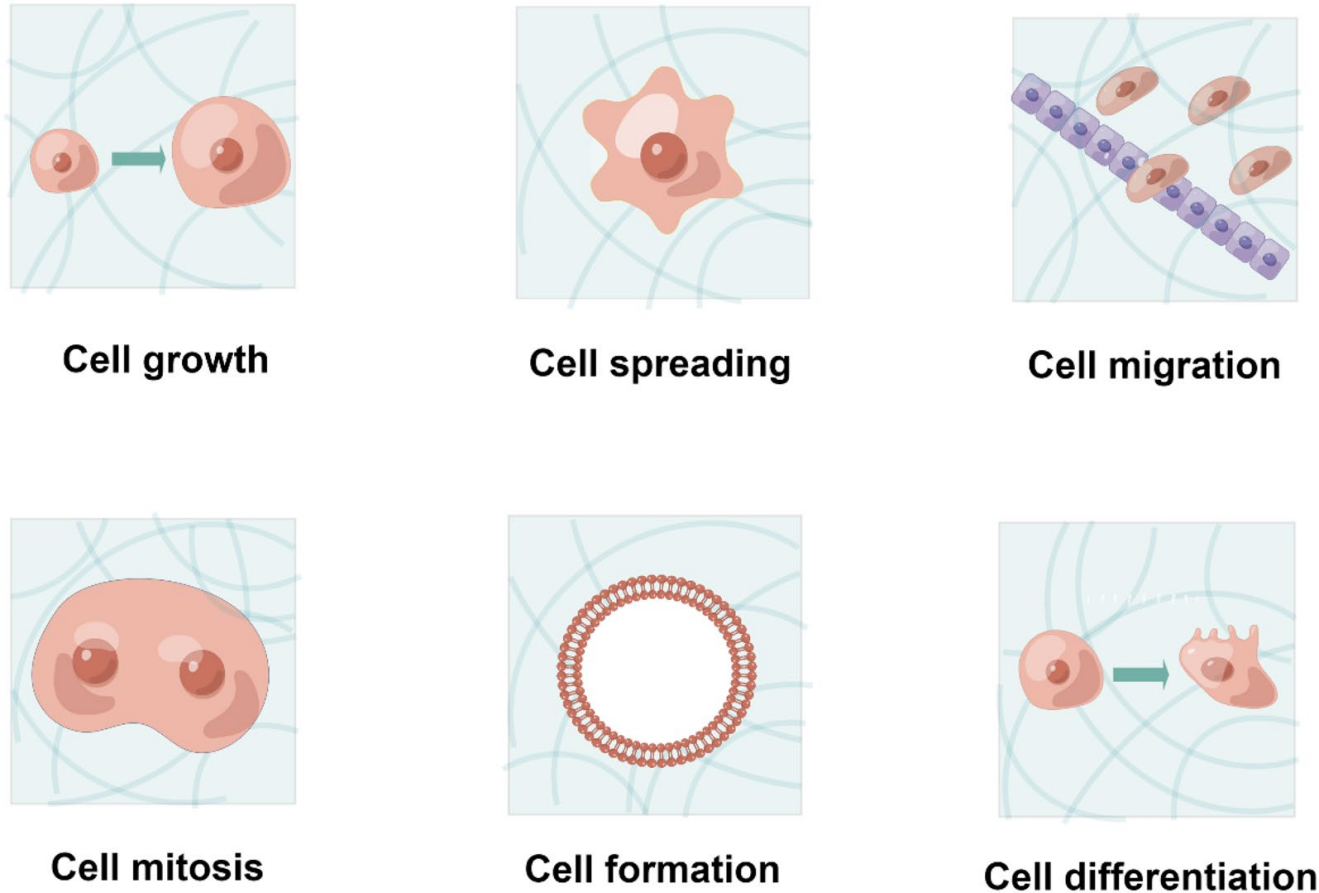
Hydrogels affect the behaviors of cells. By developing cells in hydrogels with different mechanical properties, the behaviors can be changed, including cell growth, spreading, migration, mitosis, formation, and differentiation. Created by figdraw

Tumor growth and progression depend on its surrounding microenvironment. It has become increasingly recognized that tumor microenvironment factors are among the major regulators of tumor growth and metastasis (Quail and Joyce 2013; Zhang et al. 2023b; Higginbottom et al. 2023). The ECM can also provide mechanical support, microenvironment modulation, and a source of signaling molecules to promote tumor growth (Huang et al. 2021). Therefore, to investigate the effects of tumor microenvironment on tumor development, substrate stiffness and ECM compositions can be manipulated by tuning synthetic materials’ biochemical and biophysical properties (Fig. 5). One widely used material is hydrogels, but the hydrogels used in tumor organoids have yet to be extensively studied. In this part, we conclude the application of hydrogel in tumor organoids with the aim of providing new ideas for future studies.

#### Breast cancer organoids

Several studies have demonstrated that stiffness is crucial to breast cancer development and migration (Yue et al. 2018; Colombo and Multicellular 2021). Tumor cells can increase the stiffness of the ECM, which can also influence drug treatment (Pérez-González et al. 2021). Previous research discovered that breast cancer could modify ECM, which was most likely caused by an altered phenotype of adipose progenitor cells, but this has not been identified in 3D cell culture (Sorrentino et al. 2020; Manivong et al. 2023). A recent study identified stiffness in 3D models by encapsulating aggressive tumor organoids with or without co-culturing breast cancer cells. Encapsulation of two different aggressive tumor organoids in low-stiffness hydrogel (0.5 kPa) was substantially less inhibited than in high-stiffness hydrogel (1.5 kPa) (Yue et al. 2018). This may be due to the fact that stiffness can inhibit adipogenesis. In addition, when encapsulating breast organoids in low-stiffness hydrogel (150 Pa) or high-stiffness hydrogel (5 kPa) and exposing them to the same dosages of paclitaxel, tumors in low stiffness hydrogel showed only a modest reduction in metabolic activity compared to high stiffness hydrogel which had significantly lower apoptosis levels (Drain et al. 2021). In conclusion, stiffness not only affects tumor growth but also has a significant impact on drug therapy. Using adjustable hydrogels to study tumors of drug therapy may become a priority in the future.

#### Colorectal cancer organoid

Patient-derived cell or tissue models of cancer have become a valuable tool in cancer biology (Lai et al. 2017; Gjorevski et al. 2022; Zhou et al. 2023). However, it was not until 2019 that patient-derived cells, or tissue were first used in a 3D cell model to study colorectal cancer, as reported by Shengyong Ng et al. (Ng et al. 2019). Their study created an organoid in hydrogel with adjustable stiffness (2.6, 14.6, 22.3, and 34.0 kPa) using a xenograft model taken from a colorectal cancer patient. They found organoids grew larger in 14.6 kPa hydrogel than in other stiffness levels. To validate their findings, organoids were isolated from different tumors and cultured in either 2.6 kPa or 14.6 kPa hydrogel, and it was discovered that organoids performed better or as well in 2.6 kPa hydrogel. These results suggest that hydrogel with 2.6 kPa stiffness may provide an ideal mechanical environment for culturing patient-derived cells or tissue for further research in colorectal cancer.

In this part, we conclude that hydrogel with adjustable stiffness is used in organoid development and tumor organoid. However, the current methods of studying ECM stiffness have certain limitations. For instance, heart organoids, lung organoids, and osteosarcoma organoids are not yet available, and as a result, the hydrogels with adjustable stiffness are only studied in certain organoids. Nevertheless, it is expected that adjustable hydrogels may expand beyond their present boundaries and allow the incorporation of additional organoids. Moreover, we have concluded the most appropriate stiffness for the growth of each organoid and provided different stiffness values for investigating various organoid behaviors. However, the exact mechanism by which stiffness influences the behaviors of certain organoids still needs to be fully understood. Therefore, future research can explore the specific mechanism by developing organoids in the stiffness, as mentioned above values.

## Application of hydrogels with adjustable viscoelasticity in organoids

**Fig. 6:**
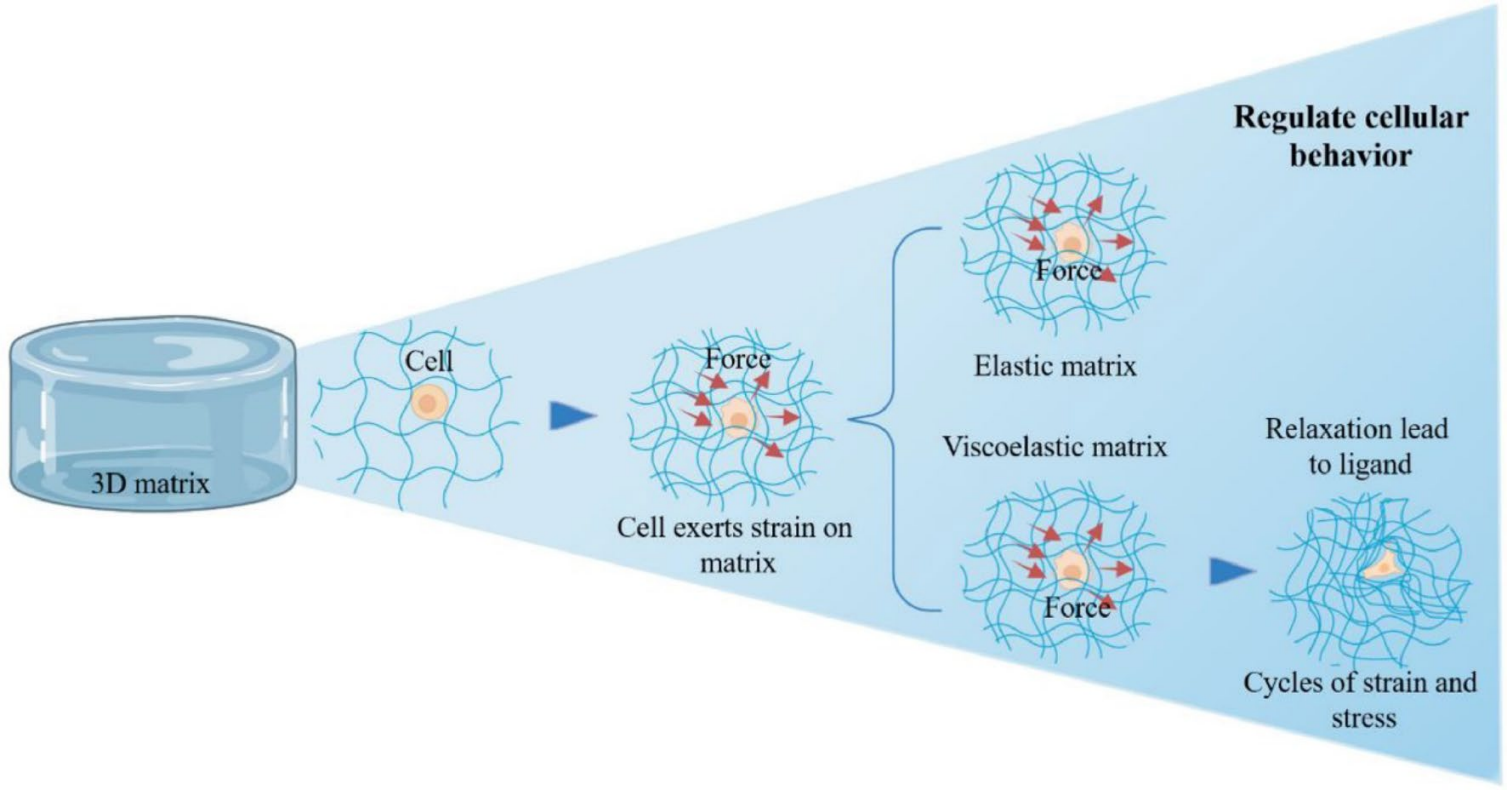
A cell exerts strain on the surrounding matrix, generating forces. In an elastic matrix, these forces remain constant, limiting matrix remodeling. In a viscoelastic matrix, stress relaxes over time, allowing for matrix remodeling and regulation of cellular behavior through cycles of strain and stress. The image is from Wu et al. (2023) and is reproduced with permission. Created by figdraw

The mechanical properties of the ECM are known to be altered during development and in various pathologies, such as cancer and fibrosis (Bonnans et al. 2014). Viscoelastic materials exhibit a time-dependent deformation response that is not instantaneous and irreversibly deforms under force (Huang et al. 2019). Although limited articles on viscoelasticity exist, synthesizing various hydrogels with viscoelastic properties may provide new sights for future research. Recent studies have highlighted the impact of viscoelasticity on biological processes, including cell migration, differentiation, and transcription factor activity (Golebiowska and Nukavarapu 2022). Owing to the current constrained utilization of viscoelastic hydrogels in organoids and the paucity of hydrogel varieties adept at nurturing organoids, we have diligently compiled the extant applications of viscoelastic hydrogels in organoid research (Fig. 6).

### The synthesis of hydrogel

Previous studies have investigated hydrogels with varying stiffness and viscoelasticity. To achieve adjustable viscoelasticity, several methods have been explored, including altering the concentration of alginate and natural-derived hydrogels, as well as using synthetic polymers (Crispim and Ito 2021; Indana et al. 2021). While recent advancements in synthetic polymers, such as PEG-based hydrogels, have demonstrated potential for tuning the viscoelastic properties of hydrogels, their large-scale application in organoid culture remains limited. However, hydrogels with adjustable viscoelasticity remain uncommon in organoid research. Tish section provides a detailed overview of hydrogel synthesis with adjustable viscoelasticity and outlines techniques for controlling them.

#### Alginate hydrogel

Alginate, a marine polysaccharide derived from brown algae and certain genetically modified bacterial species, holds promise for bone tissue engineering. However, its poor mechanical properties have been a limitation (Guo et al. 2021; McKinney et al. 2022). To address this, researchers have incorporated functional nanoparticles like HAp (Iglesias-Mejuto and García-González 2021), bioactive glass (Rottensteiner et al. 2014), metal (Raghav et al. 2022; Xu et al. 2020; Yang et al. 2020b) into alginate hydrogels. For instance, Piyachat et al. (Chuysinuan et al. 2021) developed an injectable HAp-compound fibroin-alginate hydrogel with improved mechanical strength. Additionally, combining alginate with other natural (Gilarska et al. 2021; Klara et al. 2022) or artificial biomaterials (Chen et al. 2019) has shown potential for accelerating bone regeneration. A RGD-grafted alginate/chitosan composite hydrogel exhibited highly effective bone formation and vascularization (Singh et al. 2014). Ganesh et al. (Ingavle et al. 2019) designed a composite hydrogel using alginate and hyaluronate, leading to increased blood vessel density and bone formation in a bone defect model after 12 weeks of implantation. One of the most common methods for modulating alginate hydrogels is by controlling the molecular weight of alginate chains and the concentration of calcium ions used (Bertsch et al. 2023). Previous studies have shown that alginate hydrogels can self-healing following network disruption induced by ultrasound-triggered on-demand drug release (Greco et al. 2024b; Mohamadhoseini and Mohamadnia 2021). This further extends the applicability of alginate hydrogels.

#### Liver-derived hydrogel (LEM-gel)

Naturally derived hydrogels from various organs and tissues have been developed, but few have been used for in vitro organoid development (Qian et al. 2023; Vriend et al. 2022). Decellularized ECM-based hydrogels (dECM-hydrogels), naturally derived from mammalian sources, have shown promise. In 1975, Meezan et al. successfully prepared decellularized matrices from various tissues using chemical methods, which subsequently inspired the development of numerous related techniques (Meezan et al. 1975). The effectiveness of tissue decellularization depends on factors such as cell type, tissue density, thickness, and lipid content. To date, several decellularized extracellular matrix (dECM) hydrogels have been developed, including multifunctional dECM hydrogels and reactive dECM hydrogels (Zhu et al. 2023a). For example, Kim et al. constructed a functional dECM hydrogel optimized for gastrointestinal organoid culture, while Min et al. created a hydrogel derived from decellularized porcine cardiac tissue to establish a perfusable culture system mimicking the in vivo cardiac microenvironment (Kim et al. 2022; Min et al. 2024). Comprehensive proteomic analyses, including mass spectrometry and immunohistochemistry, are critical for the quantitative and qualitative identification of dECM components (http://matrisomeproject.mit.edu). When using dECM hydrogels for organoid culture systems, factors such as tissue source, species, and decellularization protocols must be carefully considered. For instance, laminin-11 is a key component of the intestinal basement membrane, while fibronectin is essential for the bile duct. Additionally, identifying the protein composition of dECM hydrogels provides accurate and comprehensive data that can facilitate control over mechanical properties. The hydrogel derived from sheep liver has been developed and characterized (Saheli et al. 2018). Specifically, the decellularized liver pieces were lyophilized and milled to create power and stir dissolution in acid to produce a pre-gel solution. Then use NaOH to induce gel solidification. As time passes, the viscoelastic modulus will increase (first: 186.7 Pa; 10 min:497.0 Pa; 50 min: 1570.5 Pa). However, achieving stable and specific viscoelasticity remains a challenge for liver-derived hydrogels and further research is needed to address this issue and broaden their applications in organoid development.

### Utilizing hydrogels with adjustable viscoelasticity for influencing organoid development

Tissues exhibit unique stiffness characteristics, and when subjected to compression, they require a period of time to recover (Hagyousif et al. 2023). In addition to stiffness, tissues also possess viscoelasticity. For example, tendons can stretch and return to their original shape over time when stretched slowly. However, when they are rapidly extended, they can further strain-stiffen and eventually rupture (Obst et al. 2018). Therefore, using 3D models to investigate viscoelasticity is beneficial for studying the mechanical properties of ECM. In this part, we conclude that incorporating hydrogels with varying degrees of viscoelasticity may be advantageous for developing organoids specific to different organs.

#### Liver organoid

With the rising prevalence of liver cirrhosis and hepatocellular carcinoma, many studies are being conducted using liver organoids to investigate the effects of different viscoelasticity on liver development (Elosegui-Artola et al. 2023). Researchers have discovered that viscoelasticity can impact organoid development and gene expression. For instance, when liver organoids were encapsulated in viscous hydrogels with different G″ values (78.3 and 202.9), it was found that the lower viscous hydrogel (78.3) restricted organoid growth and fusion, inhibiting the formation of neocartilage tissue (Crispim and Ito 2021). On the other hand, viscous hydrogels promoted organoid growth by enhancing cell proliferation, migration, and matrix deposition. Rizwan et al. identified that viscoelastic notch signaling hydrogel induced liver bile duct organoid growth and morphogenesis (Rizwan et al. 2022). Previous studies have demonstrated that increased ECM stiffness promotes hepatocellular carcinoma progression under cirrhotic conditions. Recent research has revealed that type 2 diabetes, characterized by the accumulation of AGEs, enhances the viscoelasticity of the ECM. This increase in viscoelasticity, independent of ECM stiffness, drives HCC cell proliferation and invasion through the integrin-β1–tensin-1–YAP pathway (Fan et al. 2024). In conclusion, a viscous modulus of approximately 2.3 to 5.9 kPa should be employed to develop liver organoids, and lower viscoelasticity may facilitate organoid development.

#### HiPSCs organoid

There have been extensive efforts to develop models for embryonic development using human pluripotent stem cells (hiPSCs) (Jiang et al. 2022). However, the impact of viscoelasticity on hiPSC formation has received little attention. Indana et al. (Indana et al. (2021). investigated the role of matrix viscoelasticity in lumen formation by hiPSCs. They created an elastic modulus of either 3 or 20 kPa by adjusting the calcium cross-linking density. There were no significant differences in cell survival or cluster size between gels with an elastic modulus of 3 and 20 kPa. Moreover, after seven days of incubation, comparable levels of DNA were observed, indicating similar proliferation rates. There was also no difference in apoptosis between gels with an elastic modulus of 3 and 20 kPa. These findings suggest that viscoelasticity does not affect hiPSCs viability, apoptosis, proliferation, pluripotency, or lumen formation. However, the authors found that matrix viscoelasticity is a critical regulator of lumen formation in their culture system, and further research are needed to elucidate the mechanisms underlying the impact of viscoelasticity on hiPSC lumen formation (Indana et al. 2021).

#### Bone organoid

Previous studies have highlighted viscoelasticity’s role in providing a deformation microenvironment for cell–matrix reconstruction (Lee et al. 2021). Recent works have shown its significant influence on BMSC function, including spreading, proliferation, and differentiation (Wu et al. 2022). These findings are essential for guiding biomaterial applications in bone organoid technology (Lin et al. 2021). Alginate has been used to explore the impact of viscoelasticity on cell propagation (Grolman et al. 2020) and osteogenic differentiation (Whitehead et al. 2021). For instance, Whitehead et al. (Whitehead et al. 2021) enhanced MSC spheroids’ osteogenic differentiation potential by optimizing alginate hydrogel’s mechanical properties. Viscoelastic hydrogels consistently promoted higher calcium deposition in MSC spheroids compared to elastic hydrogels. Additionally, MSC spheroids loaded with BMP-2-HA in viscoelastic gels exhibited significantly increased calcium deposition compared to spheroids in elastic alginate gels. When implanted into critical bone defects, viscoelastic hydrogels demonstrated the highest bone formation, regardless of BMP-2 presentation. Liu et al. have demonstrated that viscoelastic hydrogels of varying stiffness can modulate nucleus pulposus regeneration (Liu et al. 2024). These findings emphasize the crucial role of viscoelasticity in regulating BMSC function and osteogenic differentiation, holding promise for bone tissue engineering with biomaterial applications.

## Application

A potential application for these hydrogels with adjustable stiffness or viscoelasticity lies in designing biomaterials for regenerative medicine (Huang et al. 2017). According to test organoids in different mechanical properties, researchers have identified mechanical properties that can affect gene expression by testing organoids with different mechanical properties. Therefore, these biomaterials (hydrogels) are typically used for cell and drug delivery, to spatially organize transplanted and resident cells, and for regulation of gene expression (Nishat et al. 2022). Similarly, organoids in adjustable hydrogels can closely mimic in vivo form and function for in vitro models or for use in regenerative medicine and drug discovery (Indana et al. 2021; Huebsch et al. 2015). Additionally, developing organoids in adjustable hydrogel has created a new platform for future study. This new system will be employed to study diseases and identify therapeutic targets in vivo (Hirota et al. 2021). In recent years, many studies are aiming to search for the specific mechanisms by which ECM promotes cancer (Mondal et al. 2020). However, due to the uncertainty of ECM composition, it is challenging to study the specific mechanism of ECM’s mechanical properties (Mukherjee and Bravo-Cordero 2023). Developing organoids in the hydrogel with adjustable mechanical properties is helpful for studying the specific mechanism that promotes cancer (Lai et al. 2024). Not only are hydrogels with adjustable mechanical useful in studying disease, but they also provide a new direction for studying molecular mechanisms of mechanical properties (Mi et al. 2024).

The mechanical properties of tissues or biomaterials can significantly influence cell behavior, including cell differentiation. Many engineered surfaces and hydrogels used for cell culture are elastic, meaning that the application of force leads to deformation, and the removal of this force allows the material to return to its original shape and size (Elosegui-Artola et al. 2023). However, most natural tissue materials are viscoelastic, possessing properties of both elastic solids and viscous liquids. The mechanical characteristics of tissues play a crucial role in directing cell behavior. In human tissues, stiffness typically ranges from soft to rigid; for example, brain and lung tissues are generally more compliant than bone. Variations in tissue stiffness are closely related to cell differentiation. For instance, intestinal organoids tend to form crypt structures at 1.3 kPa, but not at 0.3 kPa. Similarly, osteogenic differentiation occurs at 5663 Pa, whereas adipogenic differentiation happens at 77 Pa. The fates of liver, brain, and neural stem cells in vitro have also been shown to depend on stiffness (Mladenović et al. 2019). Viscoelastic materials can be characterized by their stress relaxation halftime, which is the time required for the applied stress to relax by 50% (Cacopardo et al. 2022). Compared to stiffness-controlled materials, viscoelastic materials dissipate stress energy gradually during recovery. Inspired by the viscoelasticity of ECM, researchers have investigated the impact of time-dependent mechanical properties (viscoelasticity) on cell behavior. For example, in rapidly relaxing 3D hydrogels, MSCs exhibit greater spreading, proliferation, and osteogenic differentiation compared to those in slowly relaxing matrices, independent of the material’s stiffness (Das et al. 2016). Therefore, some cells may be more sensitive to stress relaxation than to stiffness.

## Challenges and outlook

In order to thoroughly figure out the effects of changes in the mechanical properties of the ECM on cell behaviors, several challenges need to be overcome. Although stiffness is the most extensively studied mechanical property, its underlying molecular mechanism remains unclear despite its demonstrated ability to significantly influence cell behavior and gene expression (Piersma et al. 2020). Another critical issue is the efficiency at which organoid cultures can be established and the hydrogel purity, as contamination with normal cells, remains a problem. Furthermore, to allow high-throughput assays, improved methods are required to decrease the time and costs of organoid generation as well as the input material needed to establish cultures. At the same time, other mechanical properties such as ductility and toughness are a better understanding of the mechanical properties affecting cell behaviors as well as the role of cellular components in the microenvironment of organoids, which are too often still poorly understood. While some in vitro approaches have been developed to study different mechanical properties, such as immunofluorescence, existing methods need further improvement (Romano et al. 2022). In addition, advancements to co-culture organoids with hydrogels still need border implementation and explorations. Lastly, the ethical implications of organoid biobanks preserving viable patient material require further considerations and may result in stronger legislative regulation in the future (Wang et al. 2023).

As a matrix for organoid culture, synthetic hydrogels also present several drawbacks. Firstly, many synthetic hydrogels require the addition of bioadhesive agents, such as cell-binding peptides, to ensure cell adhesion; otherwise, cells may fail to attach to the hydrogel, leading to cell death rather than organoid formation. Although synthetic hydrogels are relatively inexpensive, precise placement of binding peptides can significantly increase costs and necessitate material expertise. Current research indicates that alginate hydrogels may grow on unmodified surfaces; however, further investigation is required to validate this. Moreover, the degradation of hydrogels could result in some cytotoxicity (Kang et al. 2022).

Organoid technology was developed just over ten years ago (Dekkers et al. 2013). Its rapid implementation by numerous research groups worldwide led to many breakthroughs in cell and development biology and pre-clinical (cancer) research (Lee et al. 2018). The application of organoid technology in basic cancer research has provided many new experimental models and led to various discoveries. With the establishment of living organoids in hydrogels, new possibilities arise for the broader testing and development of anti-cancer drugs and the better stratification of patient cohorts (Prestwich 2008). Currently, the most commonly used tumor models include immortalized tumor cell lines and patient-derived xenograft (PDX) models. However, these models have some inevitable drawbacks. For example, immortalized cell lines often fail to represent the heterogeneity of tumors, and PDX models, while retaining some characteristics of the parent tumor, grow slowly and have a limited lifespan, restricting their further application. Organoid technology can simulate the entire process of tumor development in vitro and, through mechanical regulation, can drive cells to differentiate in various directions. This provides a new platform for studying the effects of pathogenic gene mutations. Organoid technology also excels in drug-related research; by manipulating the mechanical properties of hydrogels, it can mimic the physiological functions and characteristics of different tissues or organs. This capability offers a high-throughput drug screening and drug delivery model (Lancaster and Knoblich 2014).

## Conclusion

In this review, we summarize the current understanding of the mechanical properties of hydrogels and their role in organoid culture. We find that different mechanical properties can influence the growth and differentiation of cells. Additionally, we outline the specific effects of stiffness and viscoelastic parameters on organoid culture. Furthermore, we discuss the current applications and challenges of mechanical manipulation of hydrogels in organoid culture. It is worth noting that there are still many mechanical properties, such as stress stiffening (where extracellular matrix becomes stiffer when subjected to stress above a critical threshold), present in biological systems that are currently difficult to replicate. Therefore, our review focuses on common mechanical properties, stiffness, and viscoelasticity, in organoid culture, providing a framework for future research endeavors.

## Data Availability

No datasets were generated or analysed during the current study.
